# Immersive In‐Person Field Courses during the Pandemic: Minimizing Risk while Maximizing Efficacy

**DOI:** 10.1002/bes2.1984

**Published:** 2022-03-29

**Authors:** John McLaughlin

**Affiliations:** ^1^ 1632 Department of Environmental Sciences College of the Environment Western Washington University Bellingham Washington 98225 USA

**Keywords:** COVID‐19, field expedition, RFSN, risk management, self‐efficacy, student learning outcome, UFERN

## Abstract

Field courses can provide formative experiences that also reduce disparities in STEM education. Impacts of the ongoing COVID‐19 pandemic on‐field programs have been particularly severe, as many institutions shifted to online instruction. Some courses retained in‐person field experiences during the pandemic, and achieved high student learning outcomes. Here, I describe an approach to mitigating risk of COVID‐19 and other hazards during expedition‐based field courses, and student learning outcomes achieved using that approach. I applied comprehensive risk management to in‐person field expeditions that treated COVID‐19 as a hazard, requiring mitigation to maintain an acceptable low level of risk. Prior to broad availability of COVID‐19 vaccines, we applied a coronavirus‐free “bubble” strategy in which all participants passed a COVID‐19 PCR test immediately before departure and then avoided contact with people outside our bubble. In the future, vaccination can reduce risk further. We implemented additional safety factors to reduce risk of incidents that could require evacuation into medical facilities overloaded with COVID‐19 patients. The courses were successful: we had no infections or other serious incidents and student learning outcomes were transformative. The approach provides a model for conducting immersive field courses during the pandemic and beyond. Several field course networks are implementing similar approaches to restore valuable field education opportunities that have declined during the pandemic.

## Introduction

In 2020, the COVID‐19 pandemic drove most undergraduate courses throughout the nation to online modalities. Faculty responded with dedication and creativity to mitigate COVID‐19 challenges (Barton 2020, Browne and Mullinax 2020, Harris et al. 2020, Lashley et al. 2020, Cohn 2021). Online learning worked well for some, but many students struggled with isolation, virtual instructor interaction, and mental health issues (Parker et al. 2021). During the first six months of the pandemic, most field course instructors reported replacing field activities with online alternatives that were less student centered (Barton 2020). Most instructors considered online activities to be poor substitutes for in‐person field activities, which also reinforced historical inequities among student identities (Barton 2020). Students from underrepresented groups confirmed instructor concerns and reported disproportionate impacts from shifts to online learning (Kimbrough 2020). After two years of intermittent or fully online instruction, some faculty are raising concerns about impacts to the educational mission (Oster 2022).

Field courses and experiences have been severely curtailed during the pandemic, raising concerns about long‐term impacts on field sciences (Swing et al. 2021). Most strategies to support inclusive student success in field courses (Zavaleta et al. 2020) were constrained or prohibited by COVID‐19 safety restrictions. Some faculty adapted by teaching DIY field‐based courses where students conducted field investigations on their own, with online faculty guidance (Race et al. 2021). Students experienced increases in some factors related to STEM success (Fey et al. 2020, McKinnon 2020), but limited gains in factors involving teamwork and social interactions (Race et al. 2021).

Some faculty devised ways to teach immersive in‐person field courses while minimizing COVID‐19 risk (Lashley and McCleery 2020, RFSN 2021). These courses applied a virus‐free “bubble” approach to achieve student learning outcomes (SLOs) while maintaining low risk of COVID‐19 exposure. Results described below were drawn from course design and student outcomes observed by the author during the last year. Burgeoning networks of other instructors and institutions are applying similar approaches (O’Connell et al. 2021, RFSN 2021).

## Risk Management Approach to Course Design

I defined two design requirements for field courses under COVID‐19 and other hazards. First was the acceptable level of risk, which determined course operational boundaries. The second was the set of student learning outcomes. Next, I designed strategies to achieve SLOs within the risk boundary. Design support tools collated by Rick Curtis (2020) were particularly helpful. Those tools help instructors and program administrators assess risk and isolate sources of hazard exposure, leading to informed risk management decisions. Isolating hazard exposure reduces broad threats down to discrete problems amenable to targeted interventions. When hazard exposure can be isolated and mitigated, in‐person SLOs can be achieved safely. When hazards cannot be mitigated with available resources, course modality must be changed.

For example, the broad threat of COVID‐19 exposure during in‐person courses could be reduced to several discrete problems, such as exposure during student transport. Physical distancing requirements might require too many vehicles to transport students to distant field sites. This constraint may preclude remote field expeditions, but local field trips with individual transportation and COVID‐19 safety protocols may remain feasible. Where students live in widely dispersed locations, some SLOs can be retained by shifting to an online field course in which instructors provide virtual direction to student field activities in their locations (McKinnon 2020, Race et al. 2021).

During the pandemic, I have deployed the full range in course modalities: online DIY field courses, day trips at local field sites mitigated with face masks and physical distancing, and immersive in‐person field expeditions. The gradient in student engagement with people and place produced parallel gradients in breadth and depth of SLO achievement (Table 1).

**Table 1:** Gradients in course modality and SLOs.

Characteristic/SLO	Online (DIY) field course	In‐person, local	In‐person, expedition
Place	Differs among students	Shared, local	Shared, remote
Time	Episodic, 1–2 hours/day	Episodic, partial day	Continuous, days–weeks
Social environment	Virtual	In‐person, distanced	In‐person, close, prolonged
Mentoring	Virtual, constrained	In‐person, constrained	Intensive, comprehensive
Peer interactions	Virtual, constrained	Episodic, in‐person	Intensive, prolonged
Science skills	Virtual, limited	Experiential, constrained	Experiential, substantial
Experimental design	Virtual, mentored	In‐person, mentored	Authentic, mentored
Sci. communication	Virtual	In‐person, distanced	In‐person, mentored
Community building	Virtual, minimal	Episodic, constrained	Continuous, liminal
Sense of belonging	Virtual May overcome disabilities	Intellectual, academic	Comprehensive, liminal
Science identity	Isolated	Episodic development	Experiential, collaborative
Self‐efficacy	Individualistic	Partial skill development Scaffolded	Strong development, intensively mentored
Comfort outdoors	Limited to pre‐existing	Limited skill, experience	Mentored, potentially great
Connection to prof. Career Interest	Virtual, mentored	Scaffolded, mentored	Experiential, continuous, mentored

Notes

Some online field courses provided greater SLO achievement than the table suggests (Race et al. 2021). “Authentic” experimental design refers to research designed by students (Goodwin et al. 2021). “Liminal” community building involves new social environments in which participants can act as professionals and develop a sense of membership (Morales et al. 2020).

The paper focuses on the immersive field expedition end of the gradient because immersive field courses (1) provide greatest potential for SLO achievement (Stumpf et al. 2008, Lashley and McCleary 2020), (2) offer greatest potential reduction in disparities in student success (Bowser and Cid 2021), and (3) have been most impacted by the COVID‐19 pandemic (Swing et al. 2021). Results presented here were derived from courses involving field expeditions in remote environments, but similar outcomes can be obtained from extended periods at residential field stations (Lashley and McCleary 2020, O'Connell et al. 2021).

## Expedition‐based Field Courses

In fall 2020 I taught a riparian conservation course that culminated in a 21‐day river expedition through the Grand Canyon in December. Two co‐instructors provided essential support in instruction, operation, risk management, logistics, and student mentoring. The course curriculum was ambitious. Most recreational groups are challenged by travel through the Grand Canyon during short cold winter days, without additional academic demands. The course curriculum spanned Grand Canyon natural history, ecology, hydrology, river safety, Leave No Trace practices, Indigenous relationships, the Glen Canyon Dam Adaptive Management Program (GCDAMP), river restoration programs, and course research projects. The curriculum scope and implementation aligned with ESA's 4‐Dimensional Ecology Education framework (Klemow et al. 2019). I set the acceptable risk of COVID‐19 infection or serious injury at a level equivalent to students remaining at home. Most course SLOs required in‐person experience. For example, students needed to observe river responses to adaptive management first‐hand, they required in‐person instructor guidance to develop and refine essential skills, and they needed to work collaboratively with peers on group field projects.

In spring 2021, I taught Environmental Science (ESCI) Field Camp, a 15‐credit block of courses that emphasized authentic student field research projects. I was the sole instructor, supported by department staff in registering students, reserving vans and rafts, configuring satellite phones, and obtaining research gear. Like the Grand Canyon course, Field Camp strived to achieve ambitious SLOs that required in‐person experience. Guided by intensive instructor mentoring, student research groups conducted literature reviews to identify knowledge gaps in their topics of interest, formulated research hypotheses, designed protocols to evaluate their hypotheses, and tested their protocols at local field sites. Then, we traveled as an entire course on two ten‐day research expeditions to implement their research designs. The first expedition involved backpacking in Olympic National Park and focused on ecosystem restoration following dam removal on the Elwha River, Washington. The second used rafts to access study sites along the Wallowa, Grande Ronde, and Snake Rivers in eastern Oregon and Washington.

## Expedition Risk Management

For both programs, achieving SLOs while minimizing COVID‐19 infection risk required innovation beyond standard COVID‐19 safety protocols. The university’s standard field course COVID‐19 protocol (WWU Provost’s Office 2021), which minimized transmission risk under an assumption that everyone was potentially infectious, was impracticable. Physical distancing would have imposed obstacles to student transport, group conduct, and river travel. Wearing face masks would have been dangerous if a student fell out of a raft into a rapid.

Instead, we employed a COVID‐19‐free “bubble” strategy for each course. The university’s Student Health Center administered COVID‐19 PCR tests to all students prior to expedition departure and reported test results the next day. Negative COVID‐19 test results were required for all participants, which gave students extra incentive to maintain rigorous infection prevention practices. Students self‐quarantined between testing and expedition departure. Then we sealed our group bubble, loaded people and gear into vans, drove to the trailhead or river launch site, and embarked on group‐isolated expeditions in remote environments. We protected the bubble throughout each expedition by avoiding contact with other people, except for a required Grand Canyon National Park ranger orientation that we mitigated with face masks, physical distancing, and holding the session outdoors.

We integrated the COVID‐19 bubble strategy into a comprehensive risk management program, using Rick Curtis’ Risk Assessment and Safety Management Model (RASM; Curtis 2015). In this model, program leaders add safety factors (training, experience, protocols, judgment, and equipment) to mitigate hazards down to an acceptable risk level. For the Grand Canyon course, we included COVID‐19 as a hazard compounding the usual risks associated with a winter expedition in a remote canyon. The Grand Canyon trip coincided with the peak of the highest COVID‐19 wave as of that date, before COVID‐19 vaccines were available. Regional hospitals were filled to capacity with COVID‐19 patients. It was imperative that we avoid the need to evacuate sick or injured course participants into an overwhelmed medical system. We mitigated hazards with diverse safety factors, including instructor training in wilderness medicine and swiftwater rescue, van driver training, in‐person river safety training, pre‐trip preparation, emergency contact gear and procedures, incident protocols and reporting, student and instructor briefings, rigorous hygiene practices, on‐river communication and travel protocols, camping gear to isolate symptomatic participants, and rescue gear and expedition first aid kits stored in multiple locations (Table 2).

**Table 2:** Risk Assessment and Safety Management model (RASM; Curtis 2015) applied to the Grand Canyon course.

Equipment	Environment	People
(a) Hazard factors		
Hard/sharp gear	Cold air (December)	Inexperience
Worn gear	Cold water	Fatigue
Rafts/entrapment	Dark/short days	Fear
Propane tanks	Storms	Haste
Stoves: hot, heavy	Flash floods	Anxiety
Firepan: hot, heavy	Rapids, ≈ Class IV	Distraction
Ropes	Strong currents	Communication
Loose straps	Holes	Leadership style
	Boulder gardens	Hazard underestimate
	Pin rocks	Tight schedule
	Undercut rocks	COVID‐19 carriers
	Unstable banks	Food allergies
	River tides	Poor hygiene
	Steep slopes	Cracked skin
	Falling rocks	
	COVID‐19	
	Waterborne diseases	
	Mice/Hantavirus	
	Wildlife bites	
	Scorpion stings	
	Isolation from EMS	
	Limited satellite cover	
(b) Safety factors		
First Aid kits	Campsite choices	Protocols: river
Rescue kits	Pre‐trip scouting	Protocols: land
Throw bags	Rapid route choice	Protocol: incident
Satellite phones	Sheltered kitchen sites	Protocol: dishwash
inReach^®^ text device	Emerg. contact list	Incident reporting
Repair kits	Check‐ins with Dept.	Protocol: COVID‐19
Safety kayak		No outsider contact
Rescue PFDs		Instructor Training
Dry suits, all people		Instructor Experience
Helmets, all people		Instructor Judgment
Maps on each boat		Leadership style
Clean boat rigging		Student Training
Hypothermia pkg.		Mentoring
COVID‐19 isolation tent		Peer‐leadership
Handwash stations		Communication: river
COVID‐19 PPE		Communication: land
Group tarps		Pre‐trip briefing
Spotlight, lanterns		Safety briefing
Water filters		Environment briefing
Gear norms		Briefings, daily
		Debriefings, frequent
		Early morning starts
		Community/support

Notes

Hazard factors tend to increase risk, and safety factors reduce risk. Safety factors can be added to reduce risk to an acceptable level. For the Grand Canyon course, a winter expedition down a remote and powerful river involved exposure to multiple substantial hazards. The COVID‐19 pandemic added new hazards that compounded consequences of incidents associated with other hazards. The instructor team added safety factors to reduce risk to an acceptable level, commensurate with remaining at home. Highway travel involved additional hazards and mitigation measures, which are not listed here.

For ESCI Field Camp, I implemented similar COVID‐19 and risk management protocols during the two research expeditions. The university restricted courses to virtual modalities during the first two weeks of the term after short notice. I pivoted during those weeks to online meetings to discuss conceptual material, review scientific writing, work with students on research project development, and prepare students for expedition logistics. Student research groups practiced their sampling protocols outside of class time and adapted the protocols based on their experiences and instructor consultation. Then, we applied a COVID‐19 bubble strategy to manage risk during each of the research expeditions. Both expeditions were preceded by COVID‐19 PCR testing and self‐quarantine. Similar to the Grand Canyon course, I mitigated COVID‐19 and other hazards using RASM as outlined in Table 2. Some safety factors in Table 2 were not necessary on the Grande Ronde River due to milder spring weather and less challenging rapids. After the expeditions, we met in person to analyze data and prepare poster presentations, mitigating COVID‐19 risk with face masks, physical distancing, and more COVID‐19 testing. In the future, vaccination can provide an essential fourth mitigation measure.

In both programs, COVID‐19 was a hazard at three levels. First, COVID‐19 threatened the health of individuals if they were to become infected. Second, COVID‐19 threatened the group: if the virus propagated through the group, it could have exhausted our resources to address other hazards while isolating and caring for patients with mild symptoms. The group threat exemplified a pandemic principle stated by Curtis (2020): the COVID‐19 context can increase severity of other hazards. The programs occurred before rapid COVID‐19 antigen tests were available, so patients with symptoms of colds, influenza, or other less virulent communicable viruses would have to be treated as if they had COVID‐19. Third, COVID‐19 was a societal hazard if a sick or injured patient required evacuation into a health care system overloaded with COVID‐19 patients. We mitigated the COVID‐19 hazard at individual and group levels with a COVID‐19‐free “bubble” strategy, rigorous hygiene, and gear to isolate symptomatic participants. We mitigated the external COVID‐19 hazard by increasing safety factors to reduce risk of injury and need for evacuation.

We mitigated psychological hazards in both programs by investing time, effort, and emphasis on cultivating inclusive and supportive communities (Demery and Pipkin 2021, Hill et al. 2021). These investments began with careful review of student course applications and student health forms, which informed instructors about student backgrounds, interests, and other student context factors. Student context factors influence student experiences and interact with course design factors to determine SLO achievement (O’Connell et al. 2021). Knowledge of context factors for each student allowed instructors to adapt orientations, instruction, and mentoring to support student needs and facilitate growth. Application review was followed by establishing group norms, group‐building activities, student‐centered pre‐trip preparation meetings, place‐based instruction, gender diversity among Grand Canyon instructors, readings representing diverse perspectives, training in expedition behavior, group meals, instructor‐student and peer‐peer mentoring, regular opportunities for individual and small group contributions to expeditions, resources to surmount individual and group challenges, and daily discussions (Table 3). These measures helped students prepare for and participate fully in expeditions that were longer and more challenging than their prior experience. The interventions and the resultant community were intended to help students develop a “resilience mindset” characterized by internalized goals, belief that their goals could be realized with effort, ability and support to respond to setbacks, and learning strategies to overcome obstacles (Yeager and Dweck 2012, Bowser and Cid 2021). Inclusive pedagogy and a resilience mindset are particularly valuable to students who identify as underrepresented minorities (Morales et al. 2020, Arif et al. 2021, Bowser and Cid 2021, Cronin et al. 2021).

**Table 3:** Six factors that support inclusive student success (a‐f: belonging, …, science identity), recommendations for incorporating the factors in field courses (Table 1 in Zavaleta et al. 2020), and course‐specific implementation measures.

Recommendation	Grand Canyon riparian conservation	ESCI field camp
(a) Belonging		
Work, travel in groups/teams	Van travel, boat groups, cook teams	Van travel; research teams; hiking groups, boat groups
Group meal prep.; celebrations	Group meals; cook teams; debrief rapid running success; awards ceremony	Group meals; cook teams; awards ceremony
Group assignments	Menu planning, research teams	Research projects, presentations, reports
Time off outside classroom	Layover days; hikes; cook team rotations; social games; music around firepan	Layover days; cook team rotations; story sharing around firepan
(b) Self‐efficacy		
Facilitate research design	Limited by time constraint; future plan	Mentored research project development
Teach, experience science skills	Field data collection, several projects; rock ID; species ID; vegetation zone ID	Field sampling methods; species ID; data analysis; poster design; scientific writing
Recognize student contribution	Awards ceremony; daily debrief; research fills data gaps in GCDAMP	Feedback on research proposals, reports, presentations; debriefs; awards ceremony; research informs restoration
(c) Comfort outdoors		
Teach, model outdoor skills	LNT; River navigation; rapid scouting; rescue training; knots; gear packing	LNT; River navigation; rapid scouting; rescue training; knots; gear packing
Provide supported experienceliving, working outdoors	Instructors camp, travel with students; Instructors part of cook teams; facilitate rapid scouting, running; gear repair, blister management; safety training; daily briefings; weather storms together	Instructor camp, travel with students; facilitate rapid scouting, running; shared meals, blister management; gear repair; safety training; daily briefings
(d) Role models		
Instructors travel, work, eat withstudents	Instructors travel, work, eat with students	Instructor travel, work, eat with students
1:1 mentoring	Develop personal goals; mentor student boat operators; frequent 1:1 interactions	Mentor student boat operators; research proposal development; 1:1 interactions
(e) Communal goals, service to society		
Cooperative problem solving	Consensus on itinerary adjustments	Discuss adaptations to weather challenges; research project adaptation
Practice varied leadership skills	Expedition behavior, multiple roles; qualified student boat captains	Expedition behavior, multiple roles; Research project leadership rotation
Student‐led inquiry	Students track rock layer sequence; students identify rapid hazards	Student‐designed research projects
Links to stewardship	Research to inform GC adaptive management; Indigenous interests, roles	Research to inform river restoration; discuss Indigenous interests, roles
(f) Science identity		
Authentic research experience	Limited by time constraints	Students design, conduct, present research

Note

By design, both the Grand Canyon course and ESCI Field Camp implemented most recommendations in Zavaleta et al. (2020).

## Assessment

I assessed program effects on student development across a broad range of SLOs (Shortlidge et al. 2021). SLO assessment included cognitive and psychomotor (skills) outcomes usually emphasized in course evaluations, but I also considered affective and societal domains that receive less attention (O'Connell et al. 2020). Assessing affective and societal outcomes is important because those domains may be distinctly impacted by immersive field experiences (Shortlidge et al. 2021), their impacts may be most enduring (McConnell and van Der Hoeven Kraft 2011), and they can reduce disparities for underrepresented minorities (Zavaleta et al. 2020).

I evaluated program effectiveness and SLO achievement with several mechanisms and information sources. Risk management protocols included daily logs and incident tracking. The group field kitchen maintained close daily contact among participants, allowing instructors to monitor student health and observe symptoms of infection. I conducted daily briefings after breakfast each morning and debriefings after dinner most evenings. These briefings provided opportunity for students to ask questions, share concerns, and report progress. Daily evening discussions provided opportunity for deeper expression of student learning, in addition to introduction of new material. Instructors reviewed student writing products to assess achievement of SLOs. For the Grand Canyon course, these products included journals, essays, and data collected for group research projects. For ESCI Field Camp, writing products included literature reviews, research project proposals and multiple revisions, project abstracts, research reports in draft and final form, and research poster presentations. I also assessed SLOs for both programs by observing student field conduct, reviewing anonymous student course evaluations, and tracking students’ post‐course professional pursuits. More formal SLO assessments (e.g., Shortlidge et al. 2021) were precluded by minimal program resources, but future courses will include formal assessments as part of a field course network (RFSN 2021).

## Results

Both programs achieved risk management expectations and student learning outcomes. They also exceeded expectations with transformative outcomes that changed students’ lives.

### Grand Canyon course results

Our COVID‐19 safety protocol and other risk management measures were successful, or at least they coincided with an absence of incidents. None of the 16 participants developed COVID‐19, were exposed to COVID‐19, or manifested symptoms of other infectious diseases. There were no serious injuries or incidents, beyond minor abrasions, minor burns while cooking, and dry skin. One vehicle tire was destroyed during highway travel by debris from a semi‐truck, but driver training and skill maintained safety throughout the event. Restrictions on in‐person activity prior to sealing our COVID‐19 bubble impacted some expedition preparation, particularly river safety training and food packing. Those activities were postponed until the group arrived at the Grand Canyon. This schedule change contributed to a long day rigging rafts at the Lees Ferry launch site. Pre‐trip investment in student resilience and community building helped sustain group enthusiasm and cooperation when we gathered in‐person, and we recovered from schedule impacts quickly. All SLOs were achieved in whole or part (Table 4). Content knowledge SLOs may have been impeded by an online modality before the expedition or reduction in time allocated for content instruction to provide additional time for essential logistical preparation. Similarly, time limitations precluded student‐designed research projects, and some students may not have engaged as fully in the instructor‐designed projects. Once in‐person field experience began, SLO achievement occurred rapidly. One student summarized the contrast between online and in‐person learning as follows.

I’ve learned so much over the past couple weeks on the river, and I feel like I’ve retained much more of it than anything I’ve learned over the past few quarters of online school. I’ve felt so isolated the past year, and this trip has made me realize really how much I need and love having good people around me.

**Table 4:** Student learning outcomes and assessment, Grand Canyon course.

Student learning outcome	Sources	Result
(a) Content knowledge		
Understand Colorado River changes after dam construction	D,J,S,E	+ +
Recognize 3 riparian zones and river management effects	D,J,S,E	+
Understand GCDAMP goals, implementation, results	D,J,S,E	+
Understand invasive plant impacts on the river system	D,S,E	+
Appreciate Grand Canyon human history and relationships	D,J,S,E	+ +
Understand rapid structure and navigation	D,I	+ +
(b) Science and field skills		
Data collection in dynamic field environments	R,I	+ +
Understanding and skills in Leave No Trace practices	D,I	+ +
Proficiency in river safety protocols and practices	D,I,P	+ +
Development of outdoor skills	D,J,I,P	+ +
(c) Transferrable skills		
Improved communication skills	D,E,I	+ +
Improved collaboration skills	D,R,E,I,P	+ + +
Improved problem‐solving skills	D,R,E,I	+ +
Improved critical thinking ability	D,S,I	+ +
Expedition behavior in concept and practice	D,J,E,I,P	+ + +
(d) Nature of science		
Increased understanding of the nature of field science	D,J,S,E,I	+ +
Stronger sense of life as a scientist	D,J,S,E,I	+
(e) Personal gains		
Increased personal self‐efficacy	D,J,E,I,P	+ + +
Increased confidence in strength, stamina, agility	D,J,I	+ +
Increased comfort in field settings	D,J,S,E,I,P	+ + +
Increased grit; perseverance through challenges	D,J,E,I,P	+ + +
(f) Professional connection		
Refinement of professional goals	D,J,S,E	+ +
Greater sense of belonging in scientific community	D,J,S,E,P	+
Development of science identity	D,J,S,E,I,P	+ +
Increased scientific self‐efficacy	D,J,S,E,I,P	+ +
(g) Broader relevance		
Increased stewardship intention and behavior	D,J,S,E,I,P	+ +
Increased connection to societal issues or problems	D,J,S,E,P	+ +

Notes

Outcomes are grouped into seven categories, (a)–(g). Assessment instrument codes: (D) discussions, (J) student journals, (S) student essays, (R) research data collection, (E) student course evaluations, (I) instructor observation, (P) post‐course professional pursuits. Outcome codes: (o) no or minimal achievement; (+) moderate achievement, (+ +) high achievement, (+ + +) exceptional achievement. SLOs with moderate results were not higher primarily due to limitations on time and online modality prior to the expedition.

### ESCI field camp results

No incidents, injuries, infections, or symptoms of illness occurred during either research expedition, suggesting the COVID‐19 and risk management protocols were effective. Avoidance of other people during both expeditions prevented exposure to COVID‐19. Black bears, elk, and other large wildlife passed near research sites and camps, but those encounters were managed without incident. Incipient rowing blisters were treated and future blisters were prevented with rowing gloves. Safety training and careful adherence to communication and travel protocols helped maintain group safety during backpacking and river expeditions. All SLOs were achieved in whole or part (Table 5). The university restriction to online instruction prior to the first expedition precluded instructor participation in sampling protocol practice sessions, but we compensated with intensive protocol refinement during early days of each expedition. Similar to the Grand Canyon course, investments in student resilience and community building facilitated successful outcomes at individual and group levels.

**Table 5:** Student learning outcomes and assessment, ESCI field camp.

Student learning outcome	Sources	Result
(a) Content knowledge		
Understand research project development process	R,E	+ + +
Ability to analyze and evaluate riparian conservation issues	D,R,E	+ +
Understand riparian system changes after dam removal	D,R,E	+ +
Understand wildlife roles in ecosystem function	D,R,E	+
Understand rapid structure and navigation	D,I	+ +
(b) Science and field skills		
Proficiency in research project design and implementation	R	+ + +
Integrate multiple perspectives and kinds of information	D,R,I	+ +
Data collection in dynamic field environments	R,I	+ + +
Scientific communication ability: aural, visual, written	R,I	+ + +
Understanding and skills in Leave No Trace practices	I	+ +
Proficiency in field and river safety protocols and practices	I	+ +
Development of outdoor skills	I	+ +
(c) Transferrable skills		
Improved communication skills	R,I	+ +
Improved collaboration skills	R,I	+ + +
Improved problem‐solving skills	R,I	+ + +
Improved critical thinking ability	R,I	+ +
Expedition behavior in concept and practice	D,I	+ + +
(d) Nature of science		
Increased understanding of the nature of field science	D,E,R,I	+ +
Stronger sense of life as a scientist	D,E,I	+ +
Increased awareness of scientific ethics	D,I	+ +
(e) Personal gains		
Increased personal self‐efficacy	D,I	+ + +
Increased confidence in strength, stamina, agility	D,I	+ +
Increased comfort in field settings	D,I,P	+ + +
Increased grit; perseverance through challenges	D,I	+ + +
(f) Professional connection		
Refinement of professional goals	D,E,P	+ +
Greater sense of belonging in scientific community	D,E,P	+ +
Development of science identity	D,E,I	+ +
Increased scientific self‐efficacy	D,R,I,P	+ + +
(g) Broader relevance		
Increased stewardship intention and behavior	D,E,I,P	+ +
Increased connection to societal issues or problems	D,E,I,P	+ +
Development as informed members of society	D,E,I,P	+ +

Notes

Outcomes are grouped into seven categories, (a)–(g). Assessment instrument codes: (D) discussions, (R) research project products, (E) student course evaluations, (I) instructor observation, (P) post‐course professional pursuits. Outcome codes: (o) no or minimal achievement; (+) moderate achievement, (+ +) high achievement, (+ + +) exceptional achievement. Achievement of some content outcomes depended on student research topics.

Student SLO achievement was demonstrated by students’ research project completion and associated gains in scientific self‐efficacy. One student described the greater benefits of student‐designed research relative to instructor‐designed projects.

It is more effective to learning the process of designing and conducting a research project and writing an accompanying research paper based on the results of your project with someone there to guide you along, but not do the work for you. I cannot imagine having to learn this for the first time on my own in the professional world where it is my livelihood and reputation on the line. I was able to learn, make mistakes, go back to the drawing board, and try again without even the fear of my grade suffering.

The student also recognized the value of research collaboration through the experience.

It is liberating to not feel like you are in competition with your classmates and actually get to work with them. Problem solving and collaboration seemed productive when they were not undermined by pressure of feeling like I had to have the ‘best’ or ‘right’ answer. We quickly got to know one another on a personal and professional level … Field Camp is not an easy course. It forces you out of your comfort zone mentally and physically, but I never considered giving up. Having fellow peers, who I now consider my friends, there going through the same things as I was and everyone supporting each other gave me a sense of belonging and strength to continue with high spirits.

### Transformative student outcomes

Both programs were transformative beyond conventional academic SLOs. Many students described their experiences as life changing. Intentional instructor investments in individual mentoring and group development fostered qualities known to increase student retention, inclusive success, and long‐term professional motivation (Zavaleta et al. 2020). These qualities included sense of belonging, self‐efficacy, comfort outdoors, role model development, cooperative problem solving, stewardship and responsibility to society, and personal and professional motivation (Table 3). Student comments, writing products, and course evaluations (Tables 4 and 5) confirmed that most affective and societal SLOs were achieved through in‐person interaction with peers and instructors, in ways that would have been minimal or absent with online modalities (Table 3).

One Grand Canyon student wrote at length about affective outcomes resulting from in‐person social experiences, which restored qualities diminished during the isolation of online learning.

I can say with ease and certainty that the realities of this course far surpassed my expectations. Not only did I accomplish all of the goals I had set for myself, I achieved more than I ever thought possible over the course of 21 days on the river (and four days squeezed into a van). The river … gave me time to reflect on and reconnect with myself and the things I want to do with my life. The mutual love and support of our community brought me much‐needed comfort, confidence, and joy during a time … of hardship, isolation, and pain. Since returning home, I have felt the lasting effects of our time on the Colorado in my attitude, outlook, and motivation.

This breadth of newly obtained academic knowledge was paired with the unexpected yet monumental personal growth I achieved while on this crazy journey. When I signed up for the course, and in the months leading up to our departure, I was apprehensive about my capacity to be successful. … As the trip progressed, the fears I had subsided, and I felt so welcomed, appreciated, and wholly part of the group. I felt more like myself than I had in months, even years. The inclusive, positive nature of our community allowed me to flourish, and I’m eternally grateful for the circumstances of being in such a beautiful place with such wonderful people, which provided me with boundless confidence and clarity. … Through the amazing comradery of our group, I was reminded of who I am and what I can contribute. The canyon brought me back to that person; a strong, independent, caring, kind individual who can bring joy and strength to others. I had lost this view of myself in my doubts and dwindling self‐confidence, but it has been restored, catalyzed by the water, sand, and sun of the Grand Canyon.

I am so immensely proud of and humbled by all that I learned and accomplished over the course of our month‐long journey. I am better for having experienced everything, from freezing cold mornings, shoving numb toes into frozen neoprene socks, to the love and warmth of circled nights around the campfire. Regardless of where my life takes me, I know that I have the strength and support to achieve whatever I put my mind to.

Another Grand Canyon student described personal gains and collaborative skill development resulting from an immersive in‐person expedition.

This course has helped me to grow personally, and strengthen my interpersonal skills. Working closely with and relying on 15 other people in an often extreme environment has challenged me to be more patient, to step up to the plate more, to be vulnerable, to lean on others and be there for them to lean on me, to share what I could and to accept support from others, and so much more. This experience has allowed me to establish strong bonds with the people around me, and to be a part of a community like one I've never experienced before. Overall, this trip has been more rewarding than I could ever have imagined and I feel so grateful to have been a part of it!

These and comparable statements from other students suggest team building and other strategies to develop students’ resilience mindset were effective (Bowser and Cid 2021). Resultant student communities were so enduring that both Grand Canyon and Field Camp student cohorts continue to meet regularly long after their programs ended and after many students have graduated.

Both programs helped students develop professional connections. Field Camp students obtained post‐program employment building on knowledge and skills developed during program. Students from both programs are applying to graduate school to pursue interests they discovered during their experiences, an educational option they had not considered for themselves. This is evidence for gains in science identity and self‐efficacy. Several Grand Canyon students pursued river guide training and became river guides during the university’s summer break. Gains in confidence, self‐efficacy, comfort outdoors, and sense of belonging they achieved during the course helped them overcome obstacles almost universally experienced by women breaking into the guiding profession (Teal 1994). Similarly, a Field Camp student has applied to the National Outdoor Leadership School’s Instructor Training Program developed to increase representation among outdoor leaders of people identifying as BIPOC.

Other students wrote about in‐person field experiences leading to recognition of stewardship responsibilities that motivated future professional intentions. After describing those intentions, one student wrote “This course has given me a clearer idea of what I want from my career.”

## Discussion

The risk management approach described here worked well for immersive field courses during the COVID‐19 pandemic. It facilitated close interaction among students and instructors without any cases of COVID‐19 or other infectious diseases. SLO achievement exceeded results from online courses (Barton 2020, Race et al. 2021). Student writing products and course evaluations indicated that most affective and societal SLOs were achieved through in‐person interaction with peers and instructors, consistent with other reports (Stumpf et al. 2008, Barton 2020, Lashley and McCleery 2020, Race et al. 2021). Benefits for students were outstanding, offering models for teaching and learning in the ongoing pandemic and beyond (O’Connell et al. 2021). These benefits help to reduce disparities for underrepresented student identities in STEM (Beltran et al. 2020, Morales et al. 2020, Bowser and Cid 2021). Instructor interventions prior to and during expeditions (Table 3) cultivated group social environments that facilitated SLO achievement. Those environments supported four student qualities: comfort with field experiences, connection to field sites, confidence from team building, and capability based on mentored growth (Bowser and Cid 2021).

Extended field expeditions generated unusually supportive communities that led to transformative student outcomes. The sense of belonging in these communities, combined with achievement of challenging goals, created a “rite of passage” experience that propelled students to greater professional aspirations (Fleischner et al. 2017, Morales et al. 2020, Bowser and Cid 2021). In this context, students experienced one of the highest expressions of the human experience. They developed strong social bonds by working collaboratively to achieve common goals, often sacrificing individual comforts. This kind of experience was formerly common in much of human history, but it has become rare in Western individualistic societies (Junger 2016). Other contexts where comparable peak social experiences are known to occur include military units, elite athletic teams, remote adventure expeditions, search and rescue teams, and Indigenous communities. When participants leave these groups, they often recall the shared experiences as the richest in their lives (Junger 2016). Students can experience their full human potential through transformative field experiences (Fleischner et al. 2017). After the course ends, many strive to maintain or recreate similar social environments. In this way, effective field programs can enrich both students and society.

COVID‐19 risk management may have conferred additional benefits. The absence of any cases of colds or flu during expeditions was unusual (Curtis 2020). Pre‐expedition COVID‐19 safety protocols may have reduced participant exposure to other communicable diseases. Rigorous attention to handwashing and dishwashing during expeditions may have further reduced risk of microbe transmission. Similarly, safety factors we implemented to mitigate COVID‐19 hazard (Table 2) may have helped to avoid other injuries or incidents.

Without larger sample sizes, it is difficult to determine whether these outcomes resulted from good luck or effective hazard mitigation. Compared with the acceptable risk level equivalent to students remaining home, zero COVID‐19 cases was low. High case rates throughout the nation in December 2020 suggest some cases were likely if students traveled home or participated in less remote social activities. In the future, COVID‐19 risk can be reduced further by vaccination and rapid antigen tests administered in the field. Risk due to travel to and from expeditions requires more analysis. Vehicle travel is one of the most dangerous activities routinely conducted by many people in the United States (National Safety Council 2022). Although travel to and from expeditions may have involved longer distances than many students would have driven at home, driving risk may have been mitigated by fewer vehicles, fewer trips, and lower risk while driving on interstate highways. Similarly, risk of injury or accident due to activities at home would require more analysis, but the absence of injuries and incidents during expeditions suggests hazard mitigation may have reduced risk to the low acceptable risk level. In summary, the expedition outcomes suggest application of the RASM (Curtis 2015) with comprehensive safety factors effectively managed in‐person field expedition risk.

Several caveats and limitations apply to results presented here. First, this paper does not provide a formal assessment of student learning outcomes. Institutional resources needed to conduct formal assessments (Shortlidge et al. 2021) were lacking. Instructor and staff support for the courses were minimal relative to comparable programs (Nicolazzo 2012, Pace et al. 2017). Staffing resources were stretched further by pandemic safety protocols. Second, a bubble approach will not work in some contexts. Maintaining a closed bubble requires full student engagement, which is possible under block scheduling or expeditions during breaks in the academic calendar. It is not practical when students move regularly among multiple courses and cohorts. Third, agency restrictions on group size can create financial or logistical barriers. Many public land management agencies limit group size in remote areas, which can limit field expeditions to courses with small enrollments or require instructor resources to support multiple course sections. Fourth, inadequate institutional funding for field programs can create financial barriers for many students (Fleischner et al. 2017). Some institutions fund expedition costs, but many hold students responsible for field course operating expenses. This financial burden can become a barrier to participation, which impacts students from underrepresented groups disproportionately (Morales et al. 2020, Cronin et al. 2021). An effective alternative is to teach courses with local field trips without operating costs (Zavaleta et al. 2020), in which potentially larger groups of students wear masks, maintain physical distancing, self‐isolate when symptomatic, and participate in vaccination and COVID‐19 testing programs. This approach may not confer transformative effects of extended expeditions (Fleischner et al. 2013), but they are more accessible and provide most of the in‐person benefits that have been lacking from online courses (Table 1). Fifth, immersive field courses involve a steep learning curve for new instructors and even experienced faculty. Resources, support, and training are available from networks of field programs, including UFERN (https://ufern.net/) and RFSN (https://riverfieldstudies.com/).

Several lessons emerged from expedition‐based field courses during the pandemic. First, immersive in‐person field programs can be highly successful while maintaining low risk, but they require extra planning, preparation, and flexibility. Second, COVID‐19 compounds other hazards in ways that complicate risk management. This more complex risk environment is best managed using RASM (Curtis 2015) or a similar structured approach. Thoughtful risk management can help overcome institutional resistance. Conversely, COVID‐19 protocols can reduce risk of other infections and protect student health. Third, COVID‐19 hazard mitigations can impact program scheduling in ways that require flexibility or SLO revisions. Fourth, a COVID‐19‐free bubble strategy requires strict vigilance to protocol. Long‐term benefits derived from protecting the bubble are worth foregoing immediate benefits available in highway convenience stores and other sources of exposure. Finally, transformative outcomes of in‐person field experiences demonstrate the value of small institutions, programs, and courses in the context of pressure to cut costs by shifting to large courses and online modalities. The quality and diversity of the next generation of ecologists and human–nature relationships depend on training and inspiration that are achieved most effectively through in‐person field experiences (Swing et al. 2021).

In summary, immersive in‐person field courses during a pandemic can be successful, even transformative, while maintaining low risk of infection. A bubble approach requires considerable planning, preparation, protocol vigilance, and adaptability. It depends on application of risk management strategies, which treat infectious disease as a hazard requiring mitigation with additional safety factors. The educational values of field experience are great (Fleischner et al. 2017, Beltran et al. 2020, Swing et al. 2021), and their benefits mitigate isolation and alienation that many students have suffered during the COVID‐19 pandemic (Oster 2022). With careful risk management, immersive field courses can be taught during the pandemic when their benefits are even greater.
